# Sensory and Compositional Properties Affecting the Likeability of Commercially Available Australian Honeys

**DOI:** 10.3390/foods10081842

**Published:** 2021-08-09

**Authors:** Maddison Hunter, Jane Kellett, Kellie Toohey, Nenad Naumovski

**Affiliations:** 1 Faculty of Health, University of Canberra, Canberra, ACT 2617, Australia; jane.kellett@canberra.edu.au (J.K.); kellie.toohey@canberra.edu.au (K.T.); nenad.naumovski@canberra.edu.au (N.N.); 2 Prehabilitation, Activity, Cancer, Exercise and Survivorship (PACES) Research Group, University of Canberra, Canberra, ACT 2617, Australia; 3 Functional Foods and Nutrition Research (FFNR) Laboratory, University of Canberra, Canberra, ACT 2617, Australia; 4 Department of Nutrition-Dietetics, School of Health Science and Education, Harokopio University, 176 71 Athens, Greece

**Keywords:** commercially available honey, sensory analysis, antioxidant, physicochemical, likeability

## Abstract

Honey’s composition and appearance is largely influenced by floral and geographic origins. Australian honeys are frequently sourced from supermarkets; however, properties associated with consumer preference and likeability remain relatively unknown. The aim of this study was to complete sensory and compositional analyses on a selection of commercially available Australian honeys. Samples (*n* = 32) were analysed for visual, olfactory and taste characteristics, with overall likeability assessed by the trained sensory panel (*n* = 24; M = 12). Compositional analysis included colour intensity (mAU); phenolic content; antioxidant characteristics (DPPH, CUPRAC); and physicochemical properties (pH, viscosity, total soluble solids). There were 23 honey samples that were significantly less liked when compared to the most liked honey (*p* < 0.05). The likeability of honey was positively associated with perceived sweetness (*p* < 0.01), and it was negatively associated with crystallisation; odour intensity; waxy, chemical, and fermented smell; mouthfeel; aftertaste; sourness; bitterness and pH (All *p*’s < 0.05). The price (AUD/100 g) was not associated with likeability (*p* = 0.143), suggesting price value potentially does not influence consumer preferences. Conclusively, differences in likeability between the honey samples demonstrate that consumer perception of sampled honeys is diverse. Honey preference is primarily driven by the organoleptic properties, particularly perceived negative tastes, rather than their antioxidant capacity or phenolic content.

## 1. Introduction

Honey is a naturally produced product made from a combination of the nectar of plants and bees own secretions, which is deposited into honey comb for maturation [1]. It is primarily composed of sugars, predominantly fructose (~36%) and glucose (~30%) [2], in addition to over 200 different nutritionally relevant compounds [3]. Among these other constituents, honey includes several enzymes, vitamins, minerals, organic acids, and a range of phytochemical compounds, such as polyphenols and carotenoids [3]. The composition of honey is largely influenced by several factors, such as its botanical origins and geographic location, as well as climate and storage conditions [2].

A variety of health benefits of honey have been identified relating to honey’s antioxidant characteristics, antibacterial properties, and anti-inflammatory effects. Honey consumption was shown to increase plasma antioxidant levels in healthy humans [4,5], indicating that honey is a potentially viable nutritional source of antioxidants. Additionally, the consumption of honey has also been shown to reduce the circulating reactive oxygen species (ROS) by-products of oxidative stress in both animal [6] and human models [4]. These antioxidant characteristics of honey can be attributed to its composition, predominately its bioactive compounds, such as phenolic acids, flavonoids, and carotenoids. The antibacterial effects of honey are ascribed to its physicochemical properties (including pH and viscosity), which have the ability to prevent the growth of bacterial species [7], and the production of hydrogen peroxide as a by-product of the breakdown of glucose caused by glucose oxidase [8]. The combined effects of the antioxidant and antibacterial properties can further lead to their synergistic anti-inflammatory effects [9,10].

The global production of honey is approximately 1.2 million tons, with the average annual consumption of honey in Australia per capita averaging 0.6–0.8 kg/year [11]. Furthermore, supermarket purchases represent 70% of honey retail in Australia [12], highlighting the acceptance of commercially available honey. The majority of commercially available honeys are exposed to a variety of different treatments and processing techniques. These include straining and filtering of the honey (to remove pollen and other plant constituents), heating (liquefication to prevent crystallisation), and pasteurisation (to destroy potential pathogens) [13]. These processes commonly include heating honey to 45 °C for 8 h, followed by filtration (100 µm) [14] in order to maintain the quality and consistency of the products and for adherence to consumer expectations of the overall product [13].

The sensory evaluation of food products traditionally involves human panellists characterising, quantifying, and interpreting the properties of a particular food product [15]. Although some laboratory analysis can quantify many characteristics of a food product, sensory evaluation is often completed when a new food product is developed or when there is an interest in the consumer’s perception of an existing food product [16]. The sensory analysis of a food product represents an essential tool in determining a variety of the product’s organoleptic properties, evaluating a products quality, and assessing the consumer opinion of the product [15].

The basis of honey sensory evaluation is the description and quantification of a variety of factors relating to the perception of visual, olfactory, gustatory, and tactile characteristics [15]. Additionally, the sensory analysis of honey can provide information relating to the botanic origin of the honey and the identification of any potential defective qualities, such as crystallisation. It is also an essential process in increasing the understanding of consumer requirements, preferences, or aversions for the evaluated honey products [17].

Desirable characteristics responsible for the overall consumer preference in the selection of honey include flavour, appearance, price/value, local origin, and convenient environmentally friendly packaging [18,19,20,21]. However, whether the composition and physical properties of honey influence consumer preference is still relatively unexplored. This could occur by multiple mechanisms, including the presence of phenolic compounds that are known to produce a bitter taste sensation [22] or levels of sugar associated with the onset of crystallisation [23]. Therefore, the aim of this study was to perform an exploratory sensory analysis of a range of commercially available Australian honeys to determine the likeability and the factors that contribute to this, considering both organoleptic and compositional attributes.

## 2. Materials and Methods

### 2.1. Honey Samples

A total of 32 commercially available honey samples were purchased from various large commercial suppliers (Aldi^®^, Coles^®^, Independent Grocers Australia^®^, Woolworths^®^) across the Australian Capital Territory (ACT; Australia), with the price (AUD/100 g) recorded. Honey samples were stored in darkness at room temperature (26 ± 3 °C) following recommended guidelines [24]. All samples were blinded to researchers from their commercial packaging, and a single researcher was responsible for sample preparation and data analysis. For the analytical analysis, if honey samples were required to be diluted based on preliminary analysis and validation, it was completed with warm deionised (DI) water (<50 °C to prevent compound degradation) [24]. An alphabetised coding system was developed; however, a detailed list of the honey samples used in this research is included in Supplementary Table S1. Prior to being coded, the honeys were arranged alphabetically according to their packaging. The honeys were assigned a specific identification letter, ranging from *A* to *AF* based on their alphabetic order. These were then used for reporting. Additionally, the unblinded honeys were categorised into numerical groups based on the information on their front of label packaging; Manuka (including honeys containing Manuka) (1), Organic (2), Generic Brand (3), Floral (4), Regional (5), and otherwise unspecified Pure (6) honeys. Due to purchasing the honey samples from supermarkets and not the original producers, no further information about the honeys, including their floral origins, was known. Each honey sample is reported according to the letter provided to them and the numerical category they were assigned to.

### 2.2. Chemicals

Chemicals used in this study included Ethanol, (±)-6-hydroxy-2,5,7,8-tetramethylchromane-2-carboxylic acid (Trolox), 2,2-diphenyl-1-picrylhydrazyl (DPPH), 6-n-propylthiouracil (PROP), Ammonium Acetate, Copper (II) Chloride Dehydrate, Folin-Ciocalteu Reagent, Gallic Acid, Neocuprine, and Sodium Carbonate and were purchased from Sigma-Aldrich (Castle Hill, Sydney, Australia). The DI water was prepared using the Millipore water purification system (Millipore Australia, North Ryde, NSW, Australia).

### 2.3. Panellist Characteristics

#### 2.3.1. Panellist Recruitment and Training

The sensory analysis protocol was assessed and approved by the University of Canberra Human Research Ethics Committee (UCHREC20191651). Informed written consent was obtained from all panellists, and procedures adhered to the approved protocol. Panellists were recruited (University of Canberra) based on their willingness to participate as representatives of the general population. A total of 24 panellists (12 males) were recruited, with a mean age of 37.0 ± 10.4 years (range 20–55 years). All assessors were required to complete sensory familiarisation training based on previously described procedures [25,26,27].

#### 2.3.2. Bitter Taste Endophenotype (6-n-Propylthiouracil Test)

Panellists were required to complete a bitter taste endophenotype test using a series (*n* = 7) of known concentrations of 6-*n*-propylthiouracil (PROP) solutions (0.017–3.20 mmol/L). The bitterness sensation of each solution was ranked by panellists using a validated and labelled magnitude scale [28]. The panellists responses were classified based on the intensity/concentration categories as follows: super tasters (limit: 0.017–0.056 mmol/L), medium taster (limit: 0.180–0.560 mmol/L), and non-taster (limit: 1.80–3.20 mmol/L) [28].

#### 2.3.3. Honey Consumption Food Frequency Questionnaire

A five-part honey consumption-specific food frequency questionnaire (Honey-FFQ) was used to determine each panellist’s honey preferences and consumption habits [12,20]. The questions provided details regarding the panellist’s honey consumption over the 12 months prior to the commencement of the sensory analysis. Further information collected included the panellist’s utilisation of honey and panellist preferences between both commercial and non-commercially available honeys.

### 2.4. Honey Sensory Analysis

#### 2.4.1. Analysis Conditions

Prior to sensory analysis, panellists fasted for two hours to ensure that taste perception was not affected [17]. Furthermore, panellists were asked to refrain from the use of perfumed toiletries to reduce potential interference in the evaluation of the olfactory properties of the selected honeys. The sensory analysis was performed in a well-lit room, where panellists were isolated and were not able to see or be influenced by other assessors in their session. A maximum of seven honeys were assessed in one evaluation session according to previous recommendations, with a 30 min break required following seven honeys [17]. All sessions were completed during the late morning to early afternoon when the sensory organs were at their maximum capability for honey assessment [17].

Panellists were asked to indicate the intensity of each attribute on a 14.5 cm rating scale, where 0 cm represented the honey not possessing the attribute and 14.5 cm for the honey representing the property entirely. In between each honey sample tasted, panellists consumed a small portion of plain white bread (one slice of bread/panellist/session) and several sips of a glass of water to ensure their palate was cleansed with no honey taste remaining for the assessment of subsequent honey samples [17].

#### 2.4.2. Visual, Olfactory, and Taste Characteristics of Selected Honeys

Panellists were provided with 40 g of a de-identified honey sample in a 200 mL capacity closed glass jar (sample/volume ratio = 1/5) to determine the visual characteristics [15]. Panellists were asked to evaluate the colour intensity, texture, and the presence of crystallisation in the honey samples provided. Furthermore, olfactory characteristics, including odour intensity and odour attributes (OA) (flowery, fruity, waxy, caramelised, acidic, chemical, and fermented), were evaluated for the same samples immediately after opening the sample jars [15]. The taste characteristics were evaluated by providing panellists with a 5 g sample, and they were required to consume a minimum of 2 g of the sample using a plastic spoon, ensuring the sample covered the tongue to maximise the surface area exposure to taste receptors [17]. Panellists were asked to report perceived mouthfeel, sample aftertaste, and specific taste intensities (TI) (sweetness, sourness, bitterness, and astringency). Finally, panellists were asked to provide the overall acceptability and likeability, or preference, of each honey based on the olfactory, visual, and taste characteristics to assess consumer acceptability of the commercially available samples. Panellists assessed each honey sample a single time.

### 2.5. Antioxidant and Physicochemcial Characteristics of Selected Honeys

#### 2.5.1. Antioxidant and Total Phenolic Composition

The antioxidant scavenging capacity was determined using the 2,2-diphenyl-1-picrylhydrazyl (DPPH) assay according to Thaipong et al. [29]. The absorbance was measured at 515 nm (Multiskan Go, Thermo Scientific, Waltham, MA, USA), and the results were expressed as millimoles of Trolox equivalents (TE) per gram of honey (mmol TE/g) using the following equation (Note: M_Trolox_ = 250.29 g/mol) [29]:

$$\mathrm{Trolox}\;\mathrm{equivalent}=\left(\left(\mathrm{Inhibition}\left(\%\right)-\left(\mathrm{intercept}\right)/\mathrm{slope}\right)/M_{\mathrm{Trolox}}\right)*-1$$

The percentage of inhibition of antioxidant activity of the honey samples was determined using the following equation:

DPPH Inhibition(%)=(1−(sample Abs/reagent blank))∗100

The cupric ion reducing capacity (CUPRAC) was determined according to [30]. The absorbance was measured at 450 nm (Multiskan Go, Thermo Scientific, USA) and expressed as millimoles of Trolox equivalents per gram of honey (mmol TE/g).

The total phenolic content (TPC) was determined using the Folin–Ciocalteur method [31]. The absorbance was measured at 765 nm (Multiskan Go, Thermo Scientific, USA), and the results were expressed as milligram Gallic Acid equivalents (GAE) per gram of the sample (mg GAE/g). All assays were completed in triplicate.

#### 2.5.2. Colour Analysis

The International Commission on Illumination (CIE) L*, a*, b* colour measurements of the honey samples were determined by evenly distributing 50 g of each honey sample around an 8.5 cm diameter clear plastic petri dish [32]. These values provide information regarding the honey’s lightness (L*; 99 = white, 0 = black), redness (+a*)/greenness (−a*), and yellowness (+b*)/blueness (−b*) [33]. Measurements were taken (*n* = 5) against a white background using a colorimeter (Color Reader CR-20, Konica Minolta, Tokyo, Japan). The colour intensity (ABS_450_) of the honey samples was determined by diluting each of the samples to a 50% concentration (*w*/*v*) [24]. The spectrophotometric absorbance was then determined in triplicate at 450 nm and 720 nm (Multiskan Go, Thermo Scientific, USA), with the difference between the two wavelengths reported as mAU.

#### 2.5.3. Physicochemical Properties

The pH of the undiluted honey samples was determined using a pH meter (Mettler Toledo, Port Melbourne, Australia) [34]. The total soluble solids (TSS), expressed as °Brix, was determined in 50% honey dilutions using a handheld digital refractometer (Opti Brix 54, Bellingham + Stanley, Kent, UK) [35], modifying for 50% honey dilutions to allow for equipment specifications. The viscosity of the undiluted honey samples was expressed in pascal seconds (Pa s) and was determined using a viscometer (Smart Series, FungiLab, Barcelona, Spain) with an R6 spindle at 5, 10, or 20 rpms depending on the percentage torque of the sample [36]. All samples were analysed in triplicate.

### 2.6. Statistical Analysis

Statistical analysis was completed using IBM SPSS Statistics version 25 (IBM Corp: Armonk, NY, USA). All variables were assessed for normality before the analysis was completed to determine if parametric or non-parametric methods would be required using histograms and the Shapiro-Wilk test for normality. The results of the normally distributed variables are reported as the mean ± standard deviation, and the not normally distributed variables are presented as the median (interquartile range). The mean or median were utilised in accordance with the distribution of each variable to report the visual, olfactory, and taste results of the sensory analysis. The perceived likeability of each honey sample, as determined by the panellists, was ranked in descending order based on their mean results. Using this ranked order, a Mann–Whitney U test was completed to determine the differences between the highest ranked honey and the remaining samples. Finally, due to the inclusion of non-parametric measures, a Kendall’s Tau coefficient of correlation was utilised to determine the relationships between the sensory analysis data and the in vitro laboratory data. The level of significance was defined as *p* < 0.05.

## 3. Results and Discussion

### 3.1. Panel Attributes

#### 3.1.1. Panellist’s Usual Honey Preferences and Consumption Habits

The findings of the Honey-FFQ identified that the panellists generally consume honey “less than once per month” (33%), “1–3 times per month” (21%), and “once a week” (21%). When panellists were asked to select all that apply, the most common reasons for the consumption of honey included as a marinade (54%), followed by as a spread (50%), as a topping for cereals and yoghurt (46%), and in drinks (46%). In total, 75% purchased their honey from various commercial stores, which is comparable to previous findings in an Australian population where 70% of consumers purchase their honey from supermarkets [12]. Contrastingly, when selecting all that apply, 33% also preferred to source their honey from alternative locations, such as farmers markets, with 25% only choosing honey from these alternative sources.

When purchasing honey, the panellists identified that price (71%), flavour (67%), and product origin (54%) were important factors in the selection of the products. Furthermore, 79% of panellists did not consider the brand, packaging, and familiarity to be important factors when making honey purchasing decisions. A study by Kortesniemi et al. [20] reported similar results where the familiarity of honey products was valued by 21% of panellists interviewed [20], with a separate study proposing that the familiarity of honey is further associated with the honey preference [37].

#### 3.1.2. Bitter Taste Endophenotype Evaluation

It is well established that plant polyphenols can be toxic when consumed due to their role in plant defence mechanisms and, therefore, are detected as bitter in taste to discourage consumption [38]. Polyphenols are transferred from the nectar and pollen of plants into the honey during honey production, resulting in a potential bitter taste [39]. Individuals may have a genetic predisposition to detect a bitter taste, such as that of the 6-*n*-propylthiouracil (PROP) solution [40], which would enhance the bitter taste experienced from honey consumption. The results of the bitter taste endophenotype PROP test [28] indicated that 83% of panellists were classified as non-tasters, while 17% classified as medium tasters, with no assessors in this study classifying as super tasters. Due to low number of the panellists demonstrating characteristics of the bitter taste polymorphism, there is insufficient evidence to suggest the detection of bitter taste in the honey samples during a sensory analysis.

### 3.2. Sensory Analysis

**Figure 1:**
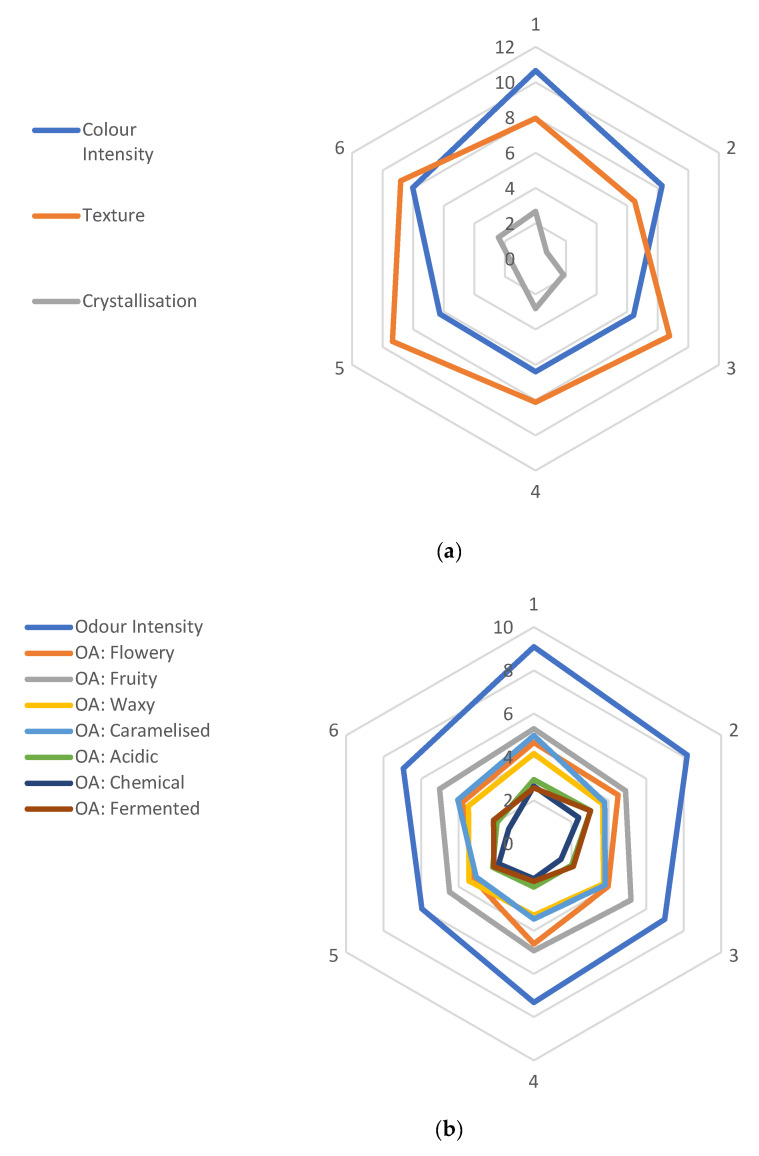

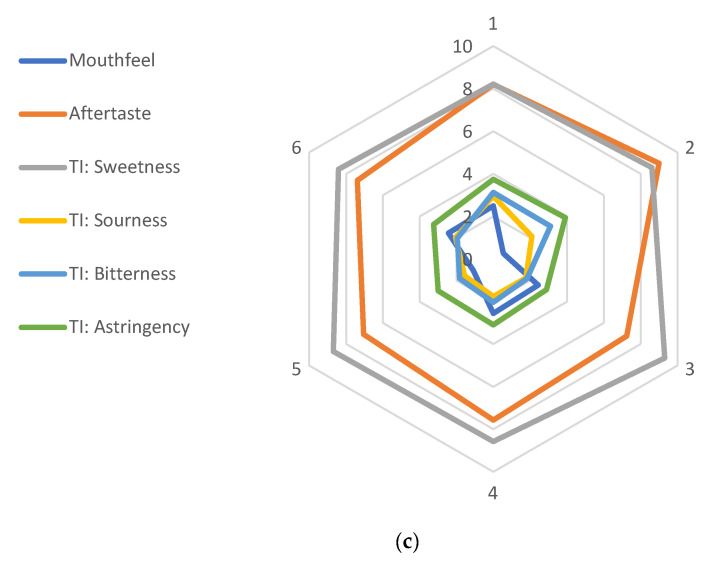
The mean values for the honey sensory analysis (honey sample *n* = 32; each sensory property assessed once). Note: (**a**) = visual characteristics; (**b**) = olfactory characteristics; (**c**) = taste characteristics. The external numbers represent the honey categories where the mean value for each sensory attribute based on panellist response (*n* = 24) is represented; 1 = Manuka Honey; 2 = Organic Honey; 3 = Generic Brand Honey; 4 = Australian Floral Honey; 5 = Regional Honey; 6 = Pure Honey. Internal numbers represent the rating scale (cm) of the sensory analysis, with each colour representing a different attribute.

The honey samples were divided into categories based on their front of label packaging, and the attributes investigated in the sensory analysis are depicted for these grouped categories (Figure 1). For the visual characteristics (Figure 1a), the colour intensity was perceived to be darkest in Manuka honeys (Category 1), with regional honeys (Category 5) containing the honeys that had the thickest texture when moved around the jar. Crystallisation was reported to be low for all categories, suggesting that the reported thick texture of the honeys was not due to the presence of crystallised sugar in the honey. This could potentially be attributed to the heat treatment processes applied to commercial honeys to prevent the resulting crystal formation from a higher sugar-to-water ratio [13].

As can be observed in Figure 1b, the individual odour attributes were not distinguishable; however, the chemical smell was least detected in these samples, with the fruity smell being the most distinct. In contrast, the odour intensity was strongly detected across all categories, particularly the Manuka category (Category 1). This suggests that the overall odour of honey is the result of a combination of the various assessed odour attributes rather than the intensity of any single odour.

The taste (Figure 1c) most strongly detected in the samples was sweetness, which can be attributed to the high sugar content of honey [2]. While the detection of the other taste characteristics was low, the aftertaste of the samples was generally high and, except for Generic Brand honey (Category 3), follows a similar pattern to the reported sweetness of the samples. Furthermore, the reported mouthfeel of the samples was low for all categories, which can be explained by the absence of crystallisation, as determined by the assessment of visual characteristics.

### 3.3. Likeability of the Honey Samples

Panellists were asked to consider the visual, olfactory, and taste properties for each of the honeys as part of the sensory analysis and report on their perceived likeability and preference for each sample (Table 1). Overall, large variations in panellist perception were observed for the range of selected honeys. For example, the smallest range observed was 9.0 cm out of a possible 14.5 cm (honey *A*) with the largest range observed being 14.5 cm (honeys *M*, *T*, and *AB*), resulting in no overall consensus for the preference of any of the honeys. Each honey is ranked in descending order in Table 1 based on their calculated means, with panellists identifying honey *C* as being the most (10.6 cm ± 2.84) and honey *E* as being the least (5.75 cm ± 3.71) liked. The completion of a Mann–Whitney U test (Table 1) allowed for the determination of the differences between honey *C* and all remaining honeys. It was identified that 23 of the remaining 31 honeys were significantly less liked than honey *C*, the most liked honey (*p* < 0.05). As anticipated, honeys with a higher mean likeability were not different from honey *C*, with the exception of honey *W* (U = 196.5, *p* = 0.059), which reported no difference despite reporting a lower mean likeability than some of the significantly different honeys.

The large ranges and significant differences in likeability of each sample suggest that the panellists did not all value the same properties of the honey. It has been identified that a variety of factors can contribute to consumer preference of honey, including texture, flavour, price, origin, and packaging [18,19,20,21]; however, with the exception of flavour and texture, the current research explores additional factors. In particular, the analysis of the sensory and in vitro properties of the honeys allowed for the identification of the specific factors that could influence consumer choice.

### 3.4. Relationships between Sensory and In Vitro Characteristics

To determine the relationships between the sensory characteristics and the in vitro antioxidant characteristics and physicochemical properties (Supplementary Table S2), a Kendall’s Tau correlation was completed. Following the determination of the ranked order for the likeability of the samples, the associations with likeability were also assessed, with a selection of the correlations presented in Table 2 (for a complete table of correlations please see Supplementary Table S3).

The likeability of the honey samples was only positively correlated with sweetness (τ = 0.353, *p* < 0.01); all other associations were negative. These included: crystallisation (τ = −0.260, *p* < 0.05), odour intensity (τ = −0.297, *p* < 0.05), the odour attributes of waxy (τ = −0.255, *p* < 0.05), chemical (τ = −0.374, *p* < 0.01), and fermented (τ = −0.324, *p* < 0.01), mouthfeel (τ = −0.288, *p* < 0.05), aftertaste (τ = −0.435, *p* < 0.01), the taste attributes of sourness (τ = −0.277, *p* < 0.05) and bitterness (τ = −0.252, *p* < 0.05), and pH (τ = −0.437, *p* < 0.01). In a relatively recent study by Cosmina et al. [18], the presence of crystals in honey were found to be disliked by Italian consumers. In addition, a preference for honeys that are more liquid in texture has been reported [18,20], supporting the data collected in this analysis. However, the preference for the mouthfeel of honey is conflicting in the literature, as a study by Murphy et al. [21] reported a preference for thick honey. Further, there were no associations between the perceived likeability and the in vitro laboratory data, except for pH. This suggests that these potential health properties did not influence the sensory characteristics of the honeys reported by panellists in this study.

In the current study, the price of honey (AUD/100 g) was not associated with the likeability (*p* = 0.143), revealing that when the price is unknown to consumers, commercially available honeys are perceived as equal. In a survey of Australian consumers that investigated participant behaviours towards honey products, the price value of honey was reported to significantly contribute to their honey selection [12], which is also supported by further studies [19,21]. The panellists in the current study did not know the value of each sample as they assessed them, and therefore, the price value of the samples could not be considered a bias towards the panellist’s overall perception of the honeys. Interestingly, the price of honey was positively associated with CUPRAC (τ = 0.271; *p* < 0.05) and TPC (τ = 0.316, *p* < 0.05), suggesting that honeys with a higher price contain higher amounts of the antioxidant-displaying compounds.

The perceived sweetness, which was associated with likeability, was inversely related to the bitter (τ = −0.271, *p* < 0.05) and sour (τ = −0.385, *p* < 0.01) tastes and a honey’s aftertaste (τ = −0.260, *p* < 0.05), which were all also negatively associated with the likeability, highlighting how important the panellists considered this taste characteristic to be. A sweet taste in food is commonly associated with its sugar content, with soluble solids also generally being correlated with sugar [2], and soluble solids comprising 80% of the sugar content [41]. Despite this, the perceived sweet taste of the samples in this study was inversely associated with the TSS (τ = −0.315; *p* < 0.05).

### 3.5. Limitations and Future Directions

A limitation of this study is that an analysis of the sugar composition of the honey samples was not completed. Determining the proportions of sugars, particularly the fructose content, could have supported the observed association between the likeability of honey and the perceived sweetness. Additionally, the honey sensory analysis was only completed once by the panellists. While all panellists were trained in this analysis, a reassessment of some of the samples would have allowed for the confirmation of the panellist’s results. Furthermore, the techniques used for some of the laboratory analysis could contribute to a limitation of the research. This includes the quantification of the phenolic content and antioxidant characteristics in this study being completed spectrophotometrically. This technology has an inability to provide detailed compositional data and can detect different compounds present at the same wavelength, resulting in the potential and inaccurate content overestimation [42].

The results acquired in this study can potentially be used to inform consumer commercial honey selection, particularly through the observed negative associations with the perceived likeability. For example, the dislike of the crystallisation could inform retailers that their honey may not be selected for a purchase in comparison to non-crystallised types. These negative associations could potentially drive consumer purchasing decisions in opposition of the selection of these honeys in comparison to the likeability of sweetness encouraging honey selection. This research was exploratory in nature. Therefore, future research should focus on completing the honey likeability assessment with a larger population size to be able to confirm the current likeability conclusions. Additionally, there should be a focus on investigating further influences on consumer honey selection, including the influence of packaging and product origin. It should also examine consumer understanding of the potential medicinal benefits of honey and if this knowledge would influence honey purchasing decisions.

## 4. Conclusions

The present study identified some of the organoleptic and physical influences on the perceived consumer likability of a range of commercially available Australian honeys. While the sweetness of honey was positively associated with the likeability, a greater range of visual, olfactory, and taste attributes, in addition to honey’s pH, were identified to be inversely correlated, which could potentially drive consumer purchasing decisions. Interestingly, neither the antioxidant profile nor retail price had an influence on the consumer perception of the honey samples, which could be due to the blinded nature of the study design and should be investigated further. Further, the differences observed between the likeability of the honey samples also demonstrate that commercial Australian honeys are not perceived to be equally preferred by the sample population of Australian consumers.

## Figures and Tables

**Table 1:** The perceived consumer likeability of a range of commercially available Australian honeys.

Rank	Honey	Minimum–Maximum (Range)	Mean ± SD	Difference	
				U	Significance
1	*C* ^4^	4.6–14.5 (9.9)	10.6 ± 2.84	-	-
2	*R* ^1^	5.1–14.5 (9.4)	9.39 ± 2.85	224.0	0.187
3	*B* ^6^	0.0–14.4 (14.4)	9.28 ± 3.96	235.5	0.279
4	*AE* ^3^	3.3–14.5 (11.2)	9.26 ± 2.84	208.0	0.099
5	*I* ^5^	2.4–14.5 (12.1)	9.13 ± 3.24	213.5	0.124
6	*Y* ^3^	2.0–14.5 (12.5)	9.09 ± 3.80	219.0	0.155
7	*J* ^6^	0.7–14.5 (13.8)	9.05 ± 3.50	217.5	0.146
8	*S* ^1^	0.8–14.5 (13.7)	8.86 ± 4.01	220.0	0.161
9	*AF* ^3^	3.5–13.7 (10.2)	8.83 ± 2.61	176.5	0.021 *
10	*D* ^5^	2.9–14.5 (11.6)	8.81 ± 2.92	181.0	0.027 *
11	*W* ^4^	2.3–14.2 (11.9)	8.70 ± 3.31	196.5	0.059
12	*V* ^6^	1.5–14.3 (12.8)	8.48 ± 3.11	179.5	0.025 *
13	*H* ^4^	2.1–14.5 (12.4)	8.46 ± 3.10	176.5	0.021 *
14	*K* ^5^	2.0–14.2 (12.2)	8.38 ± 3.49	178.5	0.024*
15	*AD* ^2^	0.0–14.4 (14.4)	8.35 ± 3.67	186.5	0.036 *
16	*O* ^6^	0.4–14.4 (14.0)	8.31 ± 3.73	178.0	0.023 *
17	*G* ^5^	0.5–13.5 (13.0)	8.26 ± 3.20	161.0	0.009 *
18	*X* ^3^	1.8–14.4 (12.6)	8.22 ± 3.54	179.5	0.025 *
19	*F* ^4^	1.8–14.1 (12.3)	8.15 ± 3.60	170.5	0.015 *
20	*L* ^3^	1.3–12.9 (11.6)	8.11 ± 3.42	170.0	0.015 *
21	*Q* ^1^	2.2–14.0 (11.8)	8.08 ± 3.26	165.0	0.011 *
22	*P* ^5^	0.4–14.1 (13.7)	7.53 ± 3.36	137.5	0.002 *
23	*N* ^4^	0.0–13.3 (13.3)	7.51 ± 3.80	158.0	0.007 *
24	*A* ^4^	3.1–12.1 (9.0)	7.45 ± 2.81	129.5	0.001 *
25	*AA* ^3^	1.3–14.4 (13.1)	7.32 ± 3.43	133.0	0.001 *
26	*U* ^1^	0.0–13.0 (13.0)	7.20 ± 3.23	122.5	0.001 *
27	*Z* ^3^	1.2–12.3 (11.1)	6.90 ± 3.02	110.5	0.000 *
28	*T* ^1^	0.0–14.5 (14.5)	6.80 ± 4.60	143.0	0.003 *
29	*M* ^1^	0.0–14.5 (14.5)	6.56 ± 4.56	138.0	0.002 *
30	*AC* ^2^	0.0–12.0 (12.0)	6.23 ± 3.76	106.5	0.000 *
31	*AB* ^4^	0.0–14.5 (14.5)	6.12 ± 4.66	133.5	0.001 *
32	*E* ^6^	0.0–12.2 (12.2)	5.75 ± 3.71	86.5	0.000 *

Note: The likeability of the honey samples (*n* = 32; likeability assessed a single time) is reported as minimum–maximum (range) and mean ± standard deviation. The superscript next to the honey identification letter represented the allocated group based on the front of label packaging description of the honey: 1 = Manuka Honey; 2 = Organic Honey; 3 = Generic Brand Honey; 4 = Australian Floral Honey; 5 = Regional Honey; 6 = Pure Honey. * Differences are significant at the 0.05 level.

**Table 2:** Kendall’s Tau correlations between a selection of the sensory attributes, antioxidant characteristics, physicochemical properties, and the price of a range of commercially available Australian honeys.

	Crystallisation	Odour Intensity	Mouthfeel	Aftertaste	Sweetness	Bitterness	Likeability	DPPH Inhibition (%)	CUPRAC	TPC	ABS_450_	pH	AUD/100 g
Crystallisation	1												
Odour Intensity	0.135	1											
Mouthfeel	0.550 **	0.148	1										
Aftertaste	−0.039	0.256 *	−0.004	1									
Sweetness	−0.176	−0.218	−0.104	−0.260 *	1								
Bitterness	0.289 *	0.262 *	0.221	0.203	−0.271 *	1							
Likeability	−0.260 *	−0.297 *	−0.288*	−0.435 **	0.353 **	−0.252 *	1						
DPPH Inhibition (%)	−0.162	0.059	−0.077	0.379 *	−0.059	0.028	−0.202	1					
CUPRAC	−0.125	0.276 *	−0.089	0.315 *	−0.131	0.118	−0.177	0.476 **	1				
TPC	−0.141	0.147	−0.077	0.234	−0.042	0.057	−0.105	0.468 **	0.677 **	1			
ABS_450_	−0.113	0.139	−0.012	0.290 *	0.006	0.061	−0.097	0.500 **	0.556 **	0.573 **	1		
pH	0.181	0.415 **	0.165	0.311 *	−0.224	0.149	−0.437 **	0.169	0.339 **	0.270 *	0.209	1	
AUD/100 g	−0.025	0.137	0.157	0.193	−0.187	0.254 *	−0.184	0.111	0.271 *	0.316 *	0.225	0.155	1

Note: Honey sample *n* = 32. Sensory properties assessed a single time, and laboratory data assessed in triplicate. ** Correlation is significant at the 0.01 level; * Correlation is significant at the 0.05 level. Further correlations in Supplementary File S3.

## Data Availability

Sensory analysis data unavailable due to ethical limitations. Laboratory data available on request from the corresponding author.

## Supplementary Materials

The following are available online at https://www.mdpi.com/article/10.3390/foods10081842/s1, Table S1: Description of the selected commercially available Australian honeys, Table S2: The antioxidant (DPPH %Inhibition, DPPH, CUPRAC), phenolic (TPC), colour (Colour Intensity (ABS_450_), *L*, a*, b**), and physicochemical (pH, TSS, Viscosity) composition of a range of commercially available Australian honeys, Table S3: Kendall’s Tau correlations between the sensory attributes, antioxidant characteristics, colour, physicochemical properties, and price for a range of commercially available Australian honeys.
