# Supplementary material for: Sensory and Compositional Properties Affecting the Likeability of Commercially Available Australian Honeys
Source: Foods. 2021 Aug 9;10(8):1842. doi: 10.3390/foods10081842 (PMC8393184; doi:10.3390/foods10081842)
Supplement: Supplementary file 1 [file foods-10-01842-s001.zip › Hunter et al_Supplementary Table 1.pdf]

Supplementary Table S1: Description of the selected commercially available Australian honeys.

Honey ID	Brand	Category
A[4]	Allowrie: Mixed Blossom Honey	Australian Floral Honey
B[6]	Archibald's: Pure Honey	Pure Honey
C[4]	Beechworth Honey: Black Box Honey	Australian Floral Honey
D[5]	Beechworth Honey: Bush Honey	Regional Honey
E[6]	Beechworth Honey: Honey and Honeycomb	Pure Honey
F[4]	Beechworth Honey: Macadamia Honey	Australian Floral Honey
G[5]	Beechworth Honey: Mountain Honey	Regional Honey
H[4]	Beechworth Honey: Orange Blossom Honey	Australian Floral Honey
I[5]	Beechworth Honey: Outback Honey	Regional Honey
J[6]	Beechworth Honey: Pure Honey	Pure Honey
K[5]	Beechworth Honey: River Honey	Regional Honey
L[3]	Black and Gold: Pure Honey	Generic Brand Honey
M[1]	Bramwells: Manuka Honey M30+	Manuka Honey
N[4]	Bramwells: Mixed Blossom Honey	Australian Floral Honey
O[6]	Bramwells: Pure Honey	Pure Honey
P[5]	Byron Bay Honey: n.s.	Regional Honey
Q[1]	Capilano: 'Beeotic' Prebiotic Honey	Manuka Honey
R[1]	Capilano: Manuka Honey	Manuka Honey
S[1]	Capilano: Manuka 5+ Active Honey	Manuka Honey
T[1]	Capilano: Manuka 10+ Active Honey	Manuka Honey
U[1]	Capilano: Manuka 15+ Active Honey	Manuka Honey
V[6]	Capilano: Pure Honey	Pure Honey
W[4]	Capilano: Yellow Box Honey	Australian Floral Honey
X[3]	Coles: Pure Honey	Generic Brand Honey
Y[3]	Coles: Red Gum Honey	Generic Brand Honey
Z[3]	Coles: White Box Honey	Generic Brand Honey
AA[3]	Dick Smith: 'OzE' Honey	Generic Brand Honey
AB[4]	Golden Nectar: Leatherwood Honey	Australian Floral Honey
AC[2]	Macro: Organic Honey	Organic Honey
AD[2]	Pure Harvest: Organic Honey	Organic Honey
AE[3]	Select: Pure Blend Honey	Generic Brand Honey
AF[3]	Select: Yellow Box Honey	Generic Brand Honey

Note: n.s. – not specified. Superscripts next to the honey identification letter are related to the front of label description category of the honey; 1 = Manuka Honey; 2 = Organic Honey; 3 = Generic Brand Honey; 4 = Australian Floral Honey; 5 = Regional Honey; 6 = Pure Honey.
