# Supplementary material for: Sensory and Compositional Properties Affecting the Likeability of Commercially Available Australian Honeys
Source: Foods. 2021 Aug 9;10(8):1842. doi: 10.3390/foods10081842 (PMC8393184; doi:10.3390/foods10081842)
Supplement: Supplementary file 1 [file foods-10-01842-s001.zip › Hunter et al_Supplementary Table 2.pdf]

Supplementary Table S2: The antioxidant (DPPH %Inhibition, DPPH, CUPRAC), phenolic (TPC), colour (Colour Intensity (ABS_{450}), *L**, *a**, *b**), and physicochemical (pH, TSS, Viscosity) composition of a range of commercially available Australian honeys.

Honey ID	DPPH (mmol TE)	DPPH % Inhibition	CUPRAC (mmol TE)[a]	TPC (mg GAE)[a]	Colour Intensity ABS_{450} (mAU)[a]	*L**[a]	*a**	*b**[a]	pH[a]	TSS (°Brix)	Viscosity (Pa s)[a]
A[4]	0.416 ± 0.029	40.6 ± 2.73	12.2 (0.643)	0.444 (0.056)	467 (2.90)	23.8 (1.90)	6.74 ± 0.666	15.1 (2.60)	4.26 (0.110)	59.3 ± 0.115	45.7 (10.2)†
B[6]	0.378 ± 0.017	36.9 ± 1.67	13.0 (0.387)	0.454 (0.002)	388 (10.9)	18.4 (3.30)	3.58 ± 0.593	8.80 (2.25)	3.92 (0.220)	58.2 ± 0.001	48.0 (1.14)†
C[4]	0.527 ± 0.016	51.2 ± 1.51	12.4 (0.379)	0.452 (0.014)	492 (13.7)	16.6 (0.900)	4.24 ± 0.114	11.6 (1.30)	3.86 (0.060)	59.3 ± 0.115	60.8 (1.40)†
D[5]	0.459 ± 0.038	44.7 ± 3.68	14.5 (1.83)	0.472 (0.050)	387 (4.90)	16.7 (1.35)	3.46 ± 0.134	10.9 (1.95)	3.93 (0.060)	58.9 ± 0.115	34.4 (0.772)†
E[6]	0.328 ± 0.020	32.1 ± 1.92	11.3 (0.449)	0.374 (0.018)	241 (5.30)	20.0 (2.15)	0.980 ± 0.327	11.0 (2.55)	3.98 (0.090)	58.6 ± 0.200	40.3 (0.996)†
F[4]	0.315 ± 0.021	30.8 ± 2.03	14.4 (0.803)	0.456 (0.037)	431 (27.1)	16.8 (0.700)	4.24 ± 0.344	11.4 (1.60)	4.14 (0.120)	60.2 ± 0.001	76.4 (2.14)†
G[5]	0.464 ± 0.046	45.1 ± 4.41	14.2 (0.656)	0.464 (0.017)	486 (13.3)	17.0 (4.35)	5.70 ± 0.640	13.6 (5.05)	3.99 (0.320)	57.7 ± 0.115	20.1 (1.57)††
H[4]	0.242 ± 0.020	23.9 ± 1.94	8.28 (1.31)	0.380 (0.024)	303 (8.90)	17.2 (2.90)	2.98 ± 0.370	9.20 (2.85)	3.87 (0.070)	59.2 ± 0.001	42.7 (1.13)†
I[5]	0.324 ± 0.030	31.7 ± 2.85	11.3 (0.159)	0.375 (0.021)	261 (4.00)	22.6 (0.850)	1.98 ± 0.455	13.3 (2.90)	3.95 (0.190)	56.8 ± 0.001	18.7 (2.25)††
J[6]	0.515 ± 0.038	50.0 ± 3.68	16.8 (0.685)	0.584 (0.011)	802 (28.3)	12.8 (1.05)	5.50 ± 0.212	6.20 (0.900)	4.13 (0.050)	58.4 ± 0.001	26.6 (1.60)††
K[5]	0.412 ± 0.073	40.2 ± 7.01	11.5 (0.190)	0.406 (0.024)	506 (16.4)	15.2 (1.50)	4.60 ± 0.412	10.2 (1.65)	3.89 (0.220)	58.4 ± 0.001	ND
L[3]	0.466 ± 0.028	45.3 ± 2.66	14.8 (0.863)	0.468 (0.052)	741 (42.1)	13.3 (0.700)	5.66 ± 0.230	7.80 (0.750)	4.24 (0.010)	56.8 ± 0.001	20.5 (2.13)††
M[1]	0.594 ± 0.047	57.6 ± 4.50	19.8 (0.803)	0.549 (0.006)	634 (29.8)	33.6 (0.800)	7.90 ± 0.212	24.7 (0.400)	4.35 (0.070)	57.4 ± 0.001	158 (1.32)†

N[4]	0.423 ± 0.034	41.2 ± 3.23	13.8 (0.896)	0.444 (0.023)	519 (16.6)	20.6 (1.60)	6.44 ± 0.351	16.7 (3.10)	4.43 (0.170)	58.5 ± 0.115	11.0 (0.275)†††
O[6]	0.379 ± 0.047	37.0 ± 4.54	12.1 (0.335)	0.387 (0.036)	303 (6.70)	17.8 (1.70)	3.06 ± 0.586	11.7 (1.30)	3.96 (0.110)	59.1 ± 0.115	ND
P[5]	0.422 ± 0.052	41.1 ± 4.98	13.2 (0.931)	0.450 (0.004)	623 (9.60)	13.5 (0.550)	4.58 ± 0.084	7.60 (0.400)	4.01 (0.100)	57.8 ± 0.001	24.1 (1.70)††
Q[1]	0.465 ± 0.016	45.3 ± 1.54	15.3 (1.48)	0.604 (0.124)	782 (27.5)	12.8 (1.15)	5.82 ± 0.653	6.70 (1.80)	4.05 (0.050)	56.6 ± 0.001	18.8 (0.661)††
R[1]	0.373 ± 0.049	36.4 ± 4.67	12.6 (1.21)	0.441 (0.008)	439 (12.7)	17.5 (0.600)	3.84 ± 0.422	13.6 (1.20)	4.31 (0.160)	56.3 ± 0.115	27.5 (0.303)††
S[1]	0.391 ± 0.014	38.2 ± 1.34	16.4 (0.457)	0.563 (0.050)	905 (77.5)	13.3 (2.00)	5.96 ± 0.780	7.20 (4.25)	4.12 (0.060)	57.7 ± 0.115	19.7 (0.841)††
T[1]	0.713 ± 0.041	69.0 ± 3.97	21.8 (1.36)	0.663 (0.078)	1196 (42.9)	11.7 (0.800)	5.66 ± 0.537	5.30 (0.800)	4.14 (0.210)	57.7 ± 0.115	20.9 (1.16)††
U[1]	0.496 ± 0.019	48.2 ± 1.79	15.5 (0.792)	0.453 (0.025)	676 (43.1)	15.4 (1.60)	7.50 ± 0.436	10.9 (2.60)	4.12 (0.060)	57.7 ± 0.115	22.3 (1.11)††
V[6]	0.360 ± 0.031	35.2 ± 2.98	11.5 (0.447)	0.309 (0.125)	432 (19.6)	14.7 (1.05)	4.14 ± 0.716	9.50 (2.10)	3.98 (0.110)	55.8 ± 0.001	21.6 (1.19)††
W[4]	0.419 ± 0.008	40.8 ± 0.81	11.8 (1.13)	0.427 (0.020)	529 (3.50)	17.8 (1.40)	6.56 ± 0.195	14.2 (2.85)	3.98 (0.270)	55.3 ± 0.115	29.5 (0.457)††
X[3]	0.207 ± 0.045	20.5 ± 4.32	13.0 (0.428)	0.489 (0.045)	478 (21.6)	16.6 (2.90)	3.68 ± 0.804	10.3 (3.70)	4.32 (0.150)	56.6 ± 0.001	24.2 (3.10)††
Y[3]	0.401 ± 0.021	39.2 ± 1.97	14.1 (0.751)	0.442 (0.013)	471 (26.6)	16.4 (2.95)	3.62 ± 0.295	8.30 (1.15)	3.74 (0.020)	58.3 ± 0.306	19.9 (1.81)††
Z[3]	0.439 ± 0.076	42.8 ± 7.30	11.4 (1.32)	0.442 (0.031)	382 (12.1)	17.7 (3.05)	2.96 ± 0.750	10.7 (3.25)	4.18 (0.200)	57.4 ± 0.001	30.7 (1.66)††
AA[3]	0.545 ± 0.058	52.9 ± 5.51	14.2 (0.757)	0.495 (0.037)	560 (29.4)	15.5 (0.700)	4.96 ± 0.555	9.30 (1.20)	4.17 (0.230)	57.4 ± 0.001	25.9 (0.993)††
AB[4]	0.596 ± 0.046	57.8 ± 4.38	15.8 (0.552)	0.483 (0.044)	576 (20.0)	15.8 (1.50)	5.34 ± 0.477	11.1 (2.45)	4.60 (0.200)	58.2 ± 0.001	41.7 (2.15)†
AC[2]	0.438 ± 0.043	42.7 ± 4.12	13.2 (0.201)	0.441 (0.049)	473 (16.9)	16.3 (3.70)	4.40 ± 0.324	7.70 (3.45)	4.12 (0.190)	57.9 ± 0.115	16.8 (0.776)††

AD[2]	0.600 ± 0.088	58.1 ± 8.46	16.0 (1.10)	0.519 (0.211)	814 (38.9)	13.0 (1.35)	7.36 ± 1.78	8.00 (2.00)	4.07 (0.150)	56.2 ± 0.001	33.0 (2.19)[†]
AE[3]	0.427 ± 0.098	41.6 ± 9.40	8.51 (0.368)	0.344 (0.025)	339 (4.50)	16.8 (4.70)	2.30 ± 0.596	10.6 (4.65)	3.82 (0.160)	56.6 ± 0.001	ND
AF[3]	0.502 ± 0.009	48.8 ± 0.83	12.2 (1.11)	0.493 (0.063)	890 (5.50)	12.6 (0.850)	6.02 ± 0.205	6.30 (0.500)	3.81 (0.050)	57.2 ± 0.001	22.6 (1.27)[††]

Note: All methods were completed in triplicate for each sample, except for L^*, a^*, b^*, which was completed in quintuplicate. Normally distributed variables are presented as mean ± standard deviation, and not normally distributed variables, identified by '[a]', are reported as median (interquartile range). Superscripts next to the honey identification letter are related to the front of label description category of the honey; 1 = Manuka Honey; 2 = Organic Honey; 3 = Generic Brand Honey; 4 = Australian Floral Honey; 5 = Regional Honey; 6 = Pure Honey. ND = No Data (due to insufficient sample volume). † = 5rpm; †† = 10rpm; ††† = 20rpm.
